# Atypical lateralization of motor circuit functional connectivity in children with autism is associated with motor deficits

**DOI:** 10.1186/s13229-016-0096-6

**Published:** 2016-07-14

**Authors:** Dorothea L. Floris, Anita D. Barber, Mary Beth Nebel, Mary Martinelli, Meng-Chuan Lai, Deana Crocetti, Simon Baron-Cohen, John Suckling, James J. Pekar, Stewart H. Mostofsky

**Affiliations:** Autism Research Centre, Department of Psychiatry, University of Cambridge, Cambridge, UK; Department of Child and Adolescent Psychiatry, the Child Study Center, New York University Langone Medical Center, New York, NY USA; Center for Neurodevelopmental and Imaging Research, Kennedy Krieger Institute, Baltimore, MD USA; Department of Neurology, Johns Hopkins School of Medicine, Baltimore, MD USA; Department of Psychiatry and Behavioral Sciences, Johns Hopkins School of Medicine, Baltimore, MD USA; Child, Youth and Family Services, Centre for Addiction and Mental Health and Department of Psychiatry, University of Toronto, Toronto, Canada; Department of Psychiatry, College of Medicine, National Taiwan University Hospital, Taipei City, Taiwan; Cambridgeshire and Peterborough NHS Foundation Trust, Cambridge, UK; National Institute of Health Research, Cambridge Biomedical Research Centre, Cambridge, UK; Behavioural and Clinical Neuroscience Institute, University of Cambridge, Cambridge, UK; Brain Mapping Unit, Department of Psychiatry, University of Cambridge, Cambridge, UK; F.M. Kirby Research Center for Functional Brain Imaging, Kennedy Krieger Institute, Baltimore, USA; Department of Radiology, Johns Hopkins School of Medicine, Baltimore, USA

**Keywords:** Autism, Lateralization, Hemispheric specialization, Intrinsic functional connectivity, Motor deficits

## Abstract

**Background:**

Atypical lateralization of language-related functions has been repeatedly found in individuals with autism spectrum conditions (ASC). Few studies have, however, investigated deviations from typically occurring asymmetry of other lateralized cognitive and behavioural domains. Motor deficits are among the earliest and most prominent symptoms in individuals with ASC and precede core social and communicative symptoms.

**Methods:**

Here, we investigate whether motor circuit connectivity is (1) atypically lateralized in children with ASC and (2) whether this relates to core autistic symptoms and motor performance. Participants comprised 44 right-handed high-functioning children with autism (36 males, 8 females) and 80 typically developing control children (58 males, 22 females) matched on age, sex and performance IQ. We examined lateralization of functional motor circuit connectivity based on homotopic seeds derived from peak activations during a finger tapping paradigm. Motor performance was assessed using the Physical and Neurological Examination for Subtle Signs (PANESS).

**Results:**

Children with ASC showed rightward lateralization in mean motor circuit connectivity compared to typically developing children, and this was associated with poorer performance on all three PANESS measures.

**Conclusions:**

Our findings reveal that atypical lateralization in ASC is not restricted to language functions but is also present in circuits subserving motor functions and may underlie motor deficits in children with ASC. Future studies should investigate whether this is an age-invariant finding extending to adolescents and adults and whether these asymmetries relate to atypical lateralization in the language domain.

**Electronic supplementary material:**

The online version of this article (doi:10.1186/s13229-016-0096-6) contains supplementary material, which is available to authorized users.

## Background

The macroscopic functional organization of the human brain is characterized by lateralized specialization of the two cerebral hemispheres for cognitive and behavioural abilities. The left hemisphere exhibits dominance for language and motor control, whereas the right hemisphere is specialized for visuospatial attention [1, 2]. This division of function has been explained as an evolutionary advantage to avoid the duplication of cognitive processing and cortical representations [3]. In line with this, we previously showed that leftward lateralization of functional motor circuit connectivity is associated with better motor performance in typically developing children [4] and others have shown that the degree of language and visuospatial lateralization relates to enhanced cognitive functioning [5, 6].

Studies converge to show that this typical pattern of hemispheric specialization is not seen in individuals with autism spectrum conditions (ASC). A consistent finding is that language-related structures such as the planum temporale [7] and inferior frontal gyrus [8] are atypically rightward asymmetric in both children and adults with ASC. These findings are corroborated by functional studies reporting atypical lateralization on linguistic tasks in individuals with ASC [9–15]. Early development of atypical language lateralization might potentially serve as a biomarker and be clinically relevant if it precedes atypical language development in children with ASC [16]. Language is not the only lateralized functional domain in which individuals with ASC exhibit deficits. Clumsiness and impaired fine motor control are among the most prevalent and earliest identifiable symptoms in individuals with ASC [17–19]. Imaging studies report atypical activation of the premotor cortex [20] and the cerebellum [21–23] during motor execution and decreased connectivity of the motor execution network [22]. However, functional studies of atypical lateralization in ASC have mainly been confined to the language domain. Only a few studies have explored lateralized activation during motor tasks in ASC during imitation [24], sequence [25] or procedural learning [26].

Motor symptoms are of considerable functional and clinical relevance in ASC, given that they are among the earliest detectable behavioural problems, preceding social and language deficits and have been found to be correlated with core social and communicative impairments in ASC [27, 28]. Indeed, the neural contributions to motor skill impairments in ASC may parallel those underlying the broader range of autistic features, leading to impaired development of skills necessary for motor, social and communicative behaviours [29]. Thus, identifying the neurobiological underpinnings of motor signs in ASC is of upmost importance in view of their potential role as biological markers of the condition.

Lateralized functions are subserved by functional coordination among regions in a network rather than by individual brain loci separately. Recent advances in neuroimaging have provided tools to measure the synchronization of cortical regions that share functional properties and thus form complex neural networks. Coherent temporal correlations in slow, spontaneous low-frequency fluctuations in the blood-oxygen-level-dependent (BOLD) signal are the basis for functional resting-state connectivity, which may reflect the intrinsic organization of the human brain [30–32]. Recent resting-state studies have confirmed the presence of lateralized brain functions and have helped to elucidate differential patterns of connectivity. Left hemisphere functions such as language and fine motor control have been suggested to be more focal preventing conduction delays and enabling rapid processing [33, 34], whereas visuospatial functions require more interhemispheric integration to represent the bilateral visual space [35]. Consistent with this, Gotts et al. [5] showed that functional lateralization differs between the two hemispheres, with left-lateralized hubs including language and motor control networks showing a bias towards intrahemispheric interactions, whereas right-lateralized hubs including those subserving attention tending to operate in a more integrative manner between the two hemispheres.

Mounting evidence suggests that the neural substrates of ASC involve atypical neural connectivity and deficient neural synchronization of multiple functional networks [36–38]. Many recent studies in ASC have moved away from task-based fMRI, instead focusing on aberrant functional connectivity of networks subserving cognitive and social functions, such as the default mode network [39–42]. To date, only a few studies have explored atypical motor circuit connectivity in ASC in association with motor performance [22, 43, 44] or atypical lateralization of resting-state (motor) networks in ASC [45, 46]. This is despite the accumulated evidence of autism-associated impairments in motor control and learning [18, 28, 47–49]. Cardinale et al. [45] reported atypical rightward lateralization of multiple functional brain networks in individuals with ASC, including language, motor and visuospatial circuits, concluding that rightward lateralization constitutes a fundamental characteristic of cerebral organization in ASC.

Here, we extend the current literature on atypical cerebral lateralization in ASC to examine the lateralization of functional motor circuit connectivity. We focus on atypical intrahemispheric functional connectivity, based on studies showing greater within-hemisphere interactions for left-lateralized functions [5] and previous studies applying a similar rationale [4, 46, 50]. We hypothesized that children with ASC would show reduced leftward or increased rightward lateralization of motor connectivity, associated with poorer performance on motor tasks.

## Methods

### Participant characteristics

Participants comprised 44 right-handed children with ASC (36 males, 8 females) and 80 right-handed typically developing children (58 males, 22 females) between 8 and 12 years of age (ASC mean = 10.23, standard deviation (SD) = 1.51, range = 8.01–12.99; controls mean = 10.15, SD = 1.08, range = 8.07–12.76) (see Additional file 1; for participant characteristics of the final sample, see Table 1). Individuals with ASC were recruited through advertisements placed within community-wide service groups, schools and hospitals, as well as from outpatient clinics at the Kennedy Krieger Institute in Baltimore. Participants with ASC received a clinical diagnosis according to the criteria of the Diagnostic and Statistical Manual of Mental Disorders–IV [51], and diagnoses were confirmed using the Autism Diagnostic Interview–Revised (ADI-R; [52]) and Module 3 of the Autism Diagnostic Observation Schedule (ADOS-G; [53]). Participants were excluded if they met any of the following criteria: (1) a history of seizures and/or traumatic brain injury; (2) a full-scale IQ (FIQ) less than 80 (in cases where the FIQ was less than 80, participants were still included if their verbal comprehension index (VCI) or perceptual reasoning index (PRI) was higher than 80), as assessed by the Wechsler Intelligence Scale for Children- fourth edition (WISC-III/IV; [54, 55]); (3) a developmental language disorder; (4) reading disability; (5) visual impairment and (6) neurologic disorder such as epilepsy. The Diagnostic Interview for Children and Adolescents, fourth edition (DICA-IV; [56]) was used to determine the presence of additional psychiatric diagnoses. Children with ASC met the following additional DICA-IV diagnoses: ADHD (16), OCD (4), specific phobia (9), generalized anxiety disorder (GAD) (4) and oppositional defiant disorder (ODD) (8). Twenty-four children with ASC were actively taking psychoactive medications, including stimulants (13), selective serotonin reuptake inhibitors (SSRI) (6), clonidine (2), risperidone (2) and lithium (2). Stimulant medications were discontinued the day prior to testing; all other medications were taken as prescribed. There was no diagnosis or family history of ASC in the typically developing control group. We included only individuals in the current sample with less than 3 mm translational and 3° rotational movement over the course of the resting scan.

**Table 1:** Participant characteristics of final sample

Characteristics	ASC (*n* = 42)			Controls (*n* = 76)			Statistics
	Mean	SD	Range	Mean	SD	Range	
Sex	34 males; 8 females	–	–	54 males; 22 females	–	–	*χ* ^*2*^(1) = 0.925, *p* = 0.336
Age^a^	10.18	(1.51)	8.01–12.99	10.16	(1.03)	8.07−12.58	
							*t* = −0.092, *p* = 0.927
Full-scale IQ^b^	104.67	(15.10)	73–141	112.89	(10.78)	85–140	*t* = 3.136, *p* = 0.003
VCI^b^	107.95	(15.53)	79–134	117.29	(12.37)	85–140	*t* = 3.353, *p* = 0.001
PRI^a^	107.45	(14.31)	79–135	109.30	(11.44)	79–133	*t* = 0.768, *p* = 0.444
Handedness^a^	86.5	(Median)	50–100	88.00	(Median)	41–100	*U* = 1590.5, *p* = 0.974
ADI-R^c^							
Social	20.62	(5.82)	10–30	–			–
Communication	15.95	(4.68)	4–25	–			–
RSB	6.64	(2.15)	3–12	–			–
ADOS-G^c^							
Communication	3.40	(1.06)	1–7	–			–
Social	7.48	(1.98)	4–12	–			–
RSB	3.05	(1.70)	0–6	–			–

*Abbreviations*: *PRI* perceptual reasoning index, *VCI* verbal comprehension index

^a^There were no significant differences between the ASC and control groups in age, sex, PRI or handedness (*p* > 0.05)

^b^The two groups significantly differed in FIQ and VCI (*p* < 0.001)

^c^Information was available for all 42 individuals with ASC

### Cognitive and behavioural measures

#### Physical and Neurological Examination of Subtle Signs

Motor skills were assessed outside the scanner using the Physical and Neurological Examination of Subtle Signs (PANESS; [57]), a battery of motor control tasks designed for children and standardized for age, sex and handedness. It is sensitive to children’s developmental changes in motor skills such as balance, coordination and speed and has adequate test-retest reliability, inter-rater reliability and internal consistency. Motor signs are quantified as *dysrhythmia* (inappropriate timing or sequencing of movements) and *overflow* (unintended and unnecessary movements) examined while performing gait, station and timed limb movements. *Gaits and station* measures are based on gait and balance assessment (gaits on heels, toes and sides of feet and tandem, standing and hopping on one foot, etc.). *Timed limb movements* are assessed during performance of repetitive and sequential movements of the hands and feet such as finger tapping, hand patting and toe tapping. *Speed*, *overflow* and *dysrhythmia* are incorporated into the *total timed score*. In addition to examining the *total gait score* and *total timed score*, the *total PANESS score* was also examined. For all three measures, better performance is associated with lower scores.

### Handedness

Handedness was assessed using the Edinburgh Handedness Inventory (EHI; [58]), a self-completed questionnaire for determining hand preference. The sample comprised only right-handed individuals with EHI scores >40.

### Structural and functional magnetic resonance imaging acquisition

All participants performed a mock scan the day before the actual scan. All individuals underwent scanning on one of two 3-T Philips scanners (2D-SENSE EPI, 8-channel head coil, SENSE acceleration = 2.0), and axially oriented volumes were acquired using T2*-weighted echo-planar imaging (field of view: 256 × 256 mm, matrix size 64 × 64, repetition time = 2500 ms, echo time = 30 ms, flip angle = 75°). Resting-state scans were acquired for 5 min and 20 s. Children were asked to stay as still as possible and fixate on a centre cross. T1-weighted high-resolution anatomical images were acquired coronally (field of view 256 × 200 mm^2^, matrix size 256 × 256, repetition time = 7.99 ms, echo time = 3.76 ms, flip angle = 8°, 1 mm isotropic voxels, slice thickness = 1 mm). These were used to generate age- and gender-matched symmetrical tissue priors.

### Image preprocessing

Functional T2*-weighted images were preprocessed using statistical parametric mapping (SPM12; Wellcome Department of Imaging Neuroscience Group, London, UK; http://www.fil.ion.ucl.ac.uk/spm). Images were slice-time corrected using the middle slice as reference slice and realigned relative to their mean. The high-resolution anatomical images were then co-registered to the functional images, segmented and normalized using a symmetrical, age- and gender-matched tissue prior generated with the Template-O-Matic toolbox [59]. The use of a symmetrical template prevents an additional introduction of anatomical asymmetries that might potentially interfere with functional asymmetries [60]. The normalization transformation was then applied to the functional images. Further steps included linear detrending at each voxel in the brain to correct for scanner drift, removal of nuisance variables such as the white matter (WM) and cerebrospinal fluid (CSF) using CompCor [61] (note that we did not use global signal regression (GSR) to avoid introduction of spurious anticorrelations in the data [62]) and six absolute and six differential motion parameters, spatial smoothing (6-mm full width at half maximum (FWHM)), and temporal band-pass filtering constraining the frequency window to 0.01–0.1 Hz. To minimise the confounding influence of micromovement, we computed the average framewise displacement (FD) (based on the median due to a non-normal distribution of movement) according to Power et al. [63] and excluded any participant with a *z* score of >2.58 [64].

### Creation of seed regions

Mostofsky et al. [22] identified the right hemisphere (RH) and left hemisphere (LH) circuits involved in motor execution during a finger-sequencing paradigm in both children with ASC and neurotypical controls between 8 and 12 years. They identified regions of interest by single-group, whole-brain random effects analyses across both groups by executing one-sample *t* tests on the individual subject’s right- and left-rest contrast images. Individual within-group whole-brain activation analyses were then run for the two groups separately identifying peak activations by group. Here, peak coordinates were derived from typical children and served as centres of seeds.

Based on the ‘left hemisphere dysfunction’ theory of autism, which states that neural impairments are lateralized to the LH in individuals with ASC, we selected peak coordinates from this LH motor circuit (see Table 2). The LH motor circuit (right-sided movement) included the left sensorimotor cortex (SMC), left thalamus (TH), left putamen (PUT), bilateral supplementary motor area (SMA)-rostral (SMA-r), bilateral SMA-dorsal (SMA-d) and right anterior cerebellum (AC) seeds [4].

**Table 2:** Motor coordinates (mm) based on Mostofsky et al. [22]

	*x*	*y*	*z*
Sensorimotor	−38	−30	51
Putamen	−30	−3	−6
Thalamus	−18	−27	9
B_SMA-d	−9	9	46
	9	9	46
B_SMA-r	−12	−1	66
	12	−1	66
Anterior cerebellum	16	−51	−25

Coordinates in MNI space

*Abbreviations*: *SMA-d* dorsal SMA, *SMA-r* rostral SMA

**Fig. 1:**
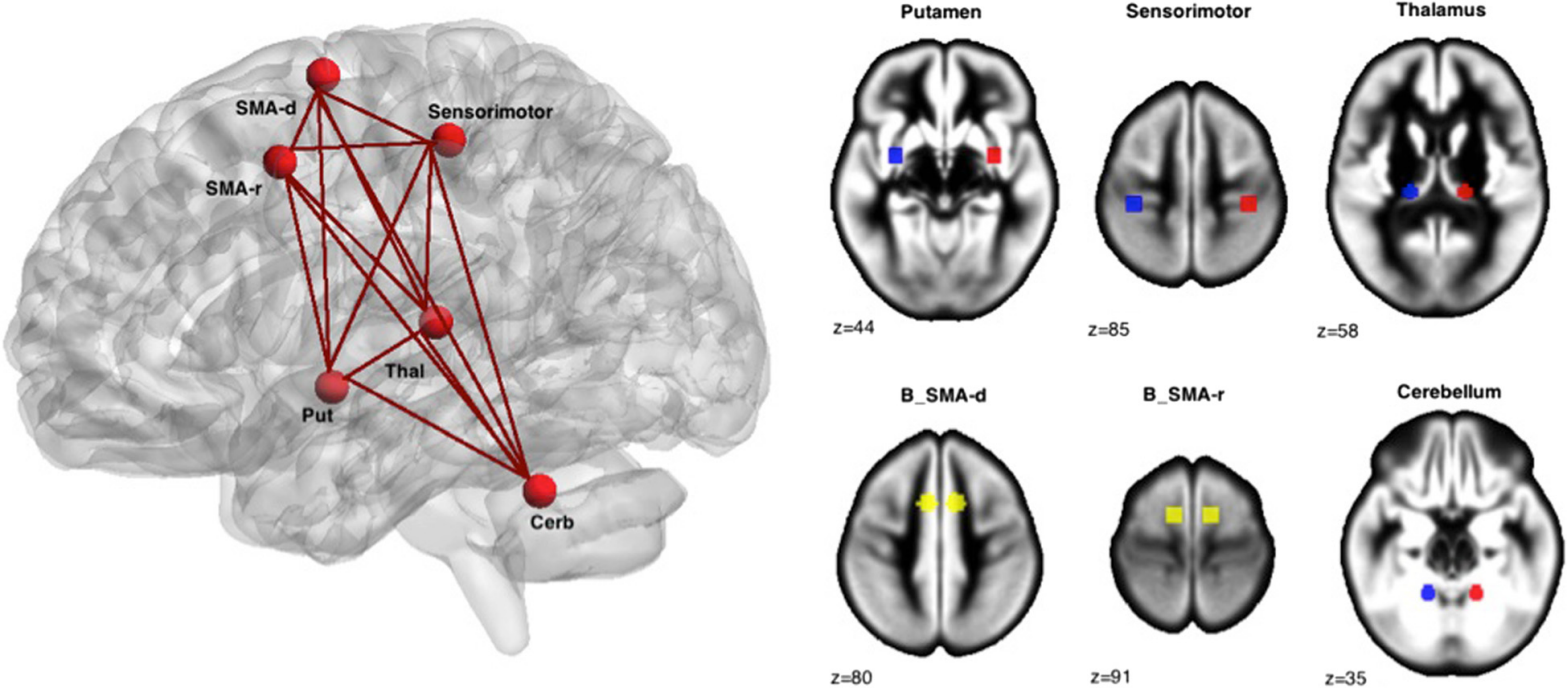
Homotopic motor seeds derived from Mostofsky et al. [22]. Abbreviations: *SMA-d* dorsal SMA, *SMA-r* rostral SMA, *Put* putamen, *Thal* thalamus, *Cerb* cerebellum. *Red*: left hemisphere seeds; *Blue* right hemisphere seeds

Six-millimetre-radius 3D seeds were then created using the SPM toolbox PickAtlas (http://fmri.wfubmc.edu/software/PickAtlas) with the peak coordinates as the centre of spheres. All seeds were flipped across the midline (*x* = 0) in order to obtain a set of homotopic RH and LH seeds (see Fig. 1). Time series were then extracted from the seed regions of interest, and pairwise Pearson’s correlations were performed for the LH and RH circuits separately. Correlation coefficients were transformed to a normal distribution via Fisher’s *z* transform. Standardized correlations between all seed pairs were then averaged to obtain mean connectivity in the motor circuit in each hemisphere (RH connectivity (*R*_con_); LH connectivity (*L*_con_)) using MATLAB. For the assessment of functional lateralization, we calculated a laterality index (LI) for each pairwise connection and for the overall mean network connectivity by subtracting the connections in the LH from homotopic connections in the right hemisphere: *R*_con_ − *L*_con_. Contrary to the usually applied formula for the calculation of structural asymmetry indices ((*R* − *L*)/(*R* + *L*)), here we did not include a denominator as functional connectivity measures comprise both positive and negative values, adversely influencing the index values [50].

### Control networks

To test whether atypical lateralization is specific to the motor network and its underlying lateralized functions, we selected two control networks: the default mode network (DMN) and a visual network (VN). DMN seeds were based on peak seed regions of the task-negative network identified by Fox et al. [65] using resting-state connectivity. Seeds were located in the cerebellum, posterior cingulate cortex (PCC), medial prefrontal cortex (MPFC), retro-splenial cortex (RSC), lateral parietal cortex (LP), superior frontal gyrus (SFG), inferior frontal gyrus (IFG) and parahippocampal gyrus (PHG). The VN seeds were based on visual seeds applied by Yeo et al. [66] and were comprised of the central and peripheral primary visual cortex (V1_c_ and V1_p_), the central and peripheral regions near the visual area V3_v_ (V3_pv_ and V3_cv_) and the extrastriate regions of the central and peripheral visual subnetworks (ExP and ExC).

### Statistical analyses

#### Group differences in mean connectivity and individual connection pairs

Participants were excluded as outliers if the average FD or laterality values had a *z* score of >2.58 [64, 67, 68]. For the analysis of the between-group differences in mean network connectivity, a univariate analysis of covariance (ANCOVA) was conducted. To compare the LIs of all the individual connectivity pairs between the two groups (ASC vs. controls), separate ANCOVAs were conducted with each individual connectivity pair as dependent variables (DV) and the global mean of the LIs of the remaining individual connectivity pairs of the network (Global-Mean_network_) as nuisance covariates in order to discount their global effect. ANCOVAs were corrected for multiple comparisons by controlling the false discovery rate (FDR) at *q* = 0.05. Age and scanning machine were included as nuisance variables in all models. To control for micromovements, average FD was included as an additional covariate [67]. Furthermore, results were re-evaluated controlling for FIQ to observe if they remained significant. To determine whether significant group differences were driven by rightward over-connectivity or leftward under-connectivity, we also compared the Fisher transformed correlation coefficients of the LH and RH circuits (*R*_con_ and *L*_con_) between the groups with the same model as that exploring the LI differences. To determine the direction of within-group circuit laterality, one-sample *t* tests were carried out. In an additional step, analyses were repeated in sex-stratified samples. Effect sizes were calculated based on Cohen’s *d*.

### Association with cognitive measures within ASC

To test whether atypical lateralization was related to function, we conducted one-tailed (based on our hypothesis that stronger rightward lateralization would be related to more symptoms and poorer performance) Pearson’s correlations controlling for scanner, age and average FD within individuals with ASC. For individual connectivity pairs, the Global-Mean_network_ was additionally included as a covariate. Motor LIs showing group differences were correlated with (a) the repetitive behaviour subscores of the ADI-R (ADI-C) and ADOS (ADOS-D) and (b) the total PANESS score, total gait score (composite measure of speed, overflow and dysrhythmia during gaits and stations) and total timed score (composite measure of speed, overflow and dysrhythmia during all timed movements). Level of significance was set at *p* = 0.05, as all correlation analyses were targeted. All statistical analyses were carried out in SPSS (version 21, SPSS Inc.).

Based on previous reports showing a linkage between motor and social development [69, 70] and the hypothesis that motor deficits might underpin some of the core social-communicative symptoms in ASC [71], an additional, exploratory analysis was conducted posing the question whether lateralization of motor circuit connectivity was related to core social and communicative symptoms, with stronger rightward lateralization being related to more deficits as measured by the ADOS-A, ADOS-B, ADI-A and ADI-B.

## Results

### Participant characteristics and task performance

Four control individuals and one individual with ASC were excluded due to *z* values of >2.58 on average FD. One additional individual with ASC was excluded due to *z* values of >2.58 on more than three laterality measures. The final sample consisted of 42 individuals with ASC (34 males, 8 females) and 76 controls (54 males, 22 females). After exclusion, the two groups were matched on age (*t*(62.51) = −0.092, *p* = 0.927), handedness (*U* = 1590.5, *z* = −0.032, *p* = 0.974), sex (*χ*^*2*^(1) = 0.925, *p* = 0.336), average FD (*U* = 1481, *z* = −0.646, *p* = 0.518) and PRI (*t*(116) = 0.768, *p* = 0.444). There were, however, significant differences in FIQ (*t*(64.87) = 3.136, *p* = 0.003) and VCI (*t*(70.10) = 3.353, *p* = 0.001). Individuals with ASC had significantly poorer performance on the total score of the PANESS (*F*(1,115) = 45.081, *p* < 0.001), and on the two sub-measures: total gait (*F*(1,115) = 36.804, *p* < 0.001) and total timed (*F*(1,115) = 32.339, *p* < 0.001).

### Group differences in lateralization of motor circuit connectivity

**Fig. 2:**
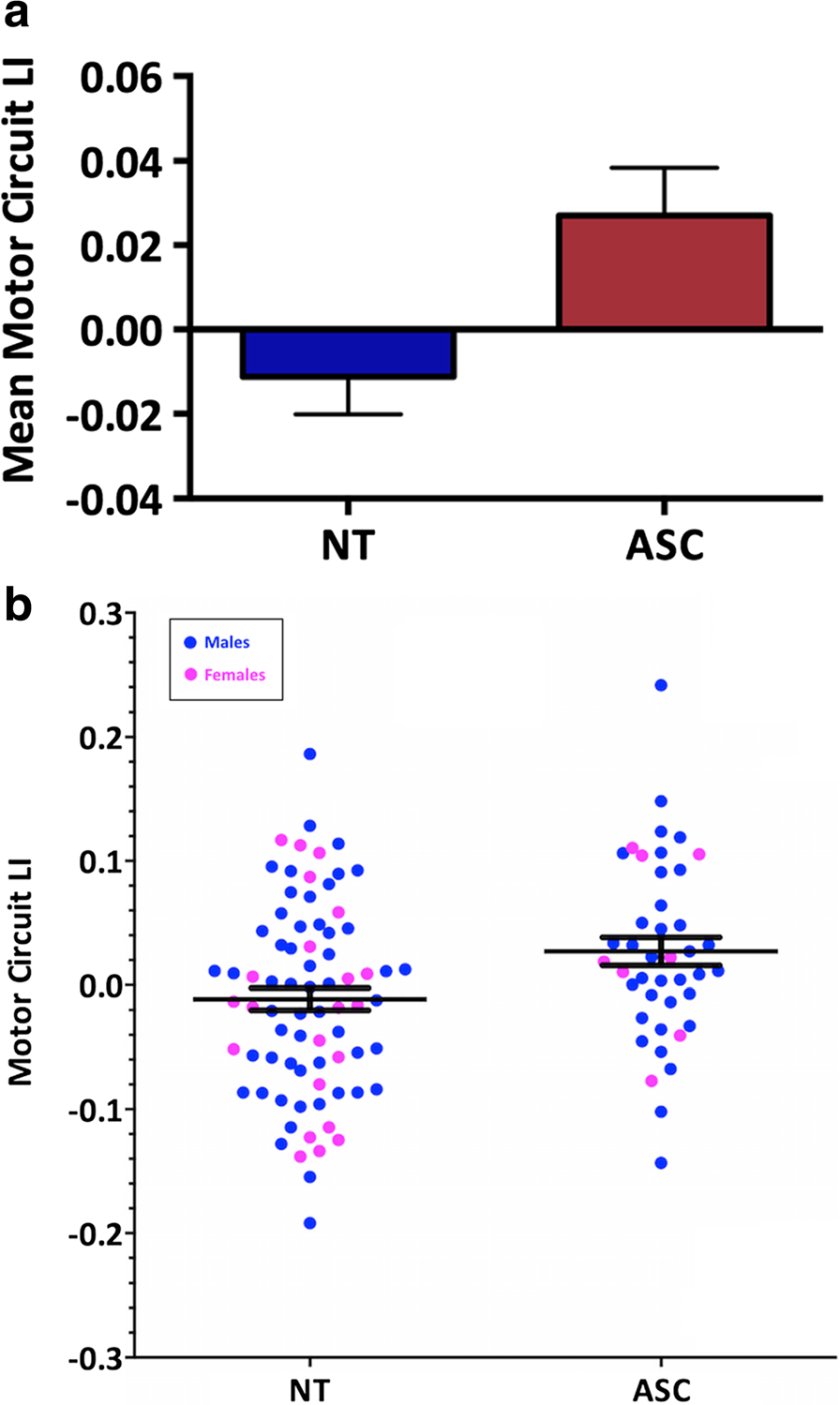
**a** Group differences in LIs of mean motor network connectivity. Abbreviations: *NT* neurotypicals (controls). Positive values indicate rightward lateralization, negative values indicate leftward lateralization. Children with ASC show reversed rightward lateralization of mean motor connectivity. **b** LIs of motor network connectivity across sexes

There was a significant difference for the LI of mean motor circuit connectivity between the ASC and control group (*F*(1,113) = 6.814, *p* = 0.010; Cohen’s *d* = 0.506), with children with ASC being more strongly rightward lateralized (see Fig. 2a). The result remained significant when controlling for FIQ (*F*(1,112) = 6.094, *p* = 0.015; Cohen’s *d* = 0.479). This difference was driven by both rightward over- and leftward under-connectivity in mean motor connectivity in children with ASC (group difference *R*_con_*F*(1,113) = 1.110, *p* = 0.294; group difference *L*_con_*F*(1,113) = 0.536, *p* = 0.466). A one-sample *t* test revealed that children with ASC were rightward lateralized (*t*(41) = 2.390, *p* = 0.022), whereas control children showed a symmetrical organization (*t*(75) = −1.255, *p* = 0.213). Individual LIs of ‘motor’ connections were not significantly different or did not survive correction for multiple comparisons (see Table 3). After stratifying analyses by sex, the result was trending in males only (*F*(1,83) = 3.920, *p* = 0.051; Cohen’s *d* = 0.438; FIQ *F*(1,82) = 2.512, *p* = 0.117; Cohen’s *d* = 0.351) and not significant in females (*F*(1,25) = 1.311, *p* = 0.263; Cohen’s *d* = 0.489; FIQ *F*(1,24) = 3.701, *p* = 0.066; Cohen’s *d* = 0.822) (for a distribution of values across sex, see Fig. 2b).

**Table 3:** Group differences in LIs of individual motor connections

Connection	ASC	Controls	*F* (1,112)	*p*	*q*
	Mean (SD)	Mean (SD)			
SMA-d-AC	0.006 (0.17)	−0.409 (0.14)	1.555	0.215	0.50
SMA-d-M1	0.072 (0.20)	0.001 (0.19)	3.756	0.055	0.35
SMA-d-Put	0.025 (0.18)	0.033 (0.17)	0.902	0.344	0.63
SMA-d-Thal	0.029 (0.19)	−0.044 (0.19)	0.583	0.447	0.63
SMA-r-AC	0.009 (0.15)	−0.026 (0.16)	0.667	0.416	0.63
SMA-r-M1	0.074 (0.22)	−0.004 (0.16)	3.126	0.08	0.35
SMA-r-Put	−0.005 (0.15)	0.049 (0.17)	7.634	0.007	0.098
SMA-r-Thal	0.003 (0.19)	−0.034 (0.18)	0.006	0.939	0.94
AC-M1	0.032 (0.22)	−0.005 (0.21)	0.161	0.689	0.80
AC-Put	0.013 (0.18)	−0.002 (0.17)	0.099	0.754	0.81
AC-Thal	0.042 (0.22)	−0.048 (0.18)	2.388	0.125	0.35
M1-Put	0.037 (0.22)	−0.009 (0.24)	0.221	0.639	0.80
M1-Thal	0.077 (0.18)	−0.019 (0.20)	2.627	0.108	0.35
Put-Thal	−0.008 (0.18)	−0.017 (0.24)	0.577	0.449	0.63

*Abbreviations*: *SMA-d* dorsal SMA, *SMA-r* rostral SMA, *SMC* sensorimotor cortex, *Put* putamen, *Thal* thalamus, *AC* anterior cerebellum

### Association with cognitive measures in children with ASC

**Fig. 3:**
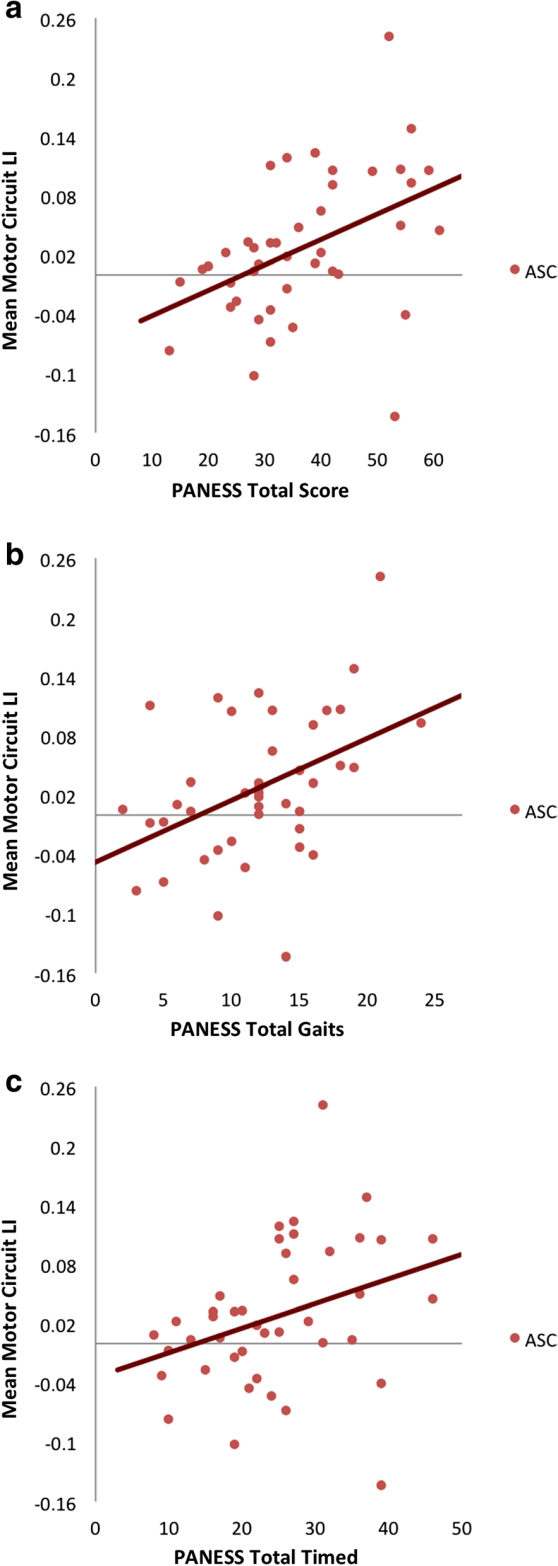
Association between LI of mean motor network connectivity and motor-related symptoms. **a** Association between LI of mean motor network connectivity and PANESS total scores. **b** Association between LI of mean motor network connectivity and PANESS total gaits scores. **c** Association between LI of mean motor network connectivity and PANESS total timed scores

The LI of total mean motor connectivity was positively associated with the total PANESS scores (*r* = 0.455, *p* = 0.002), total gait subscores (*r* = 0.434, *p* = 0.003) and total timed subscores (*r* = 0.357, *p* = 0.013) (see Table 4). In each case, stronger rightward lateralization was associated with worse performance on the motor task (see Fig. 3a–c). There were no associations with either the ADOS-D (*r* = 0.117, *p* = 0.238) or ADI-C (*r* = −0.026, *p* = 0.438). There were no significant associations between the LI of mean motor connectivity and the ADOS-A (*r* = 0.127, *p* = 0.220), ADOS-B (*r* = 0.217, *p* = 0.093), ADI-A (*r* = 0.065, *p* = 0.348) and ADI-B (*r* = −0.075, *p* = 0.325). Correlations with the PANESS were not significant in typical children (total PANESS score *r* = −0.059, *p* = 0.310; total gait subscores *r* = 0.094, *p* = 0.215; total timed subscores *r* = −0.132, *p* = 0.133).

**Table 4:** Correlations between the LI of mean motor circuit connectivity and motor-related symptoms

Motor measure	LI mean motor circuit connectivity
PANESS total	r = 0.455, p = 0.002*, q = 0.008
PANESS gait	r = 0.434, p = 0.003*, q = 0.008
PANESS timed	r = 0.357, p = 0.013*, q = 0.021
ADI-C	*r* = -0.026, *p* = 0.438, *q* = 0.438
ADOS-D	*r* = 0.117, *p* = 0.238, *q* = 0.298

* = significant at q<0.05

### Control networks

#### Default mode network

There was no significant difference in LI of mean DMN connectivity (*F*(1,113) = 2.12, *p* = 0.143) between individuals with ASC and controls. Individual LIs of DMN connections were not significantly different or did not survive correction for multiple comparisons (for details, see Additional file 1).

### Visual network

There was no significant difference in the LI of mean VN connectivity (*F*(1,113) = 0.467; *p* = 0.496) between individuals with ASC and controls. Individual LIs of VN connections were not significantly different (for details, see Additional file 1).

## Discussion

In the current study, we investigated whether the functional motor execution network shows an atypical pattern of hemispheric specialization in ASC. Consistent with our hypothesis, we found stronger rightward lateralization in children with ASC compared to typically developing children. Disturbance in motor functioning in children with ASC has been linked to dysfunction in multiple motor regions/systems, including the fronto-striatal and cerebellar systems [22, 43, 72–74]. However, few studies have explored the resting-state functional connectivity within the motor execution system in children with ASC [22, 43, 44]. Mostofsky et al. [22] report the overall under-connectivity, which is more pronounced during motor execution than during rest. Nebel et al. [44] focused specifically on M1 connectivity, examining autism-associated differences in regional M1 functional parcellation; they found organizational differentiation in the motor homunculus pointing to an early failure in functional specialization in the motor cortex in children with ASC. Here, we further show that the neurobiological underpinnings of motor impairments in ASC are related to atypical hemispheric lateralization in motor circuits.

Our finding is particularly interesting in the light of rising theories suggesting that autism is a condition of atypical connectivity involving multiple cerebral networks deficient in synchronization and information integration [36, 37]. However, so far, most functional connectivity studies in ASC have focused on under- or over-connectivity of networks critically involved in cognitive and social functions such as the default mode network [39–42] but have not addressed atypical lateralization of these circuits. Our findings are in line with Cardinale et al.’s [45] report of rightward lateralization of the motor networks. They reported on a wider range of networks, raising the question whether atypical rightward asymmetry is a more fundamental feature of network organization in ASC and not restricted to specific functions such as language and motor behaviour. However, we did not find atypical lateralization patterns across all networks; that is, we observed no group differences in the visual and default mode networks. These discrepancies might be due to differences in methodology between the two studies; Cardinale et al. [45] applied a model-free whole-brain approach aggregating information across many regions, whereas we took a hypothesis-driven approach, focusing on specific regions and their interconnections using seed-based analysis. While seed-based and ICA-based analyses yield similar results [75], using a model-free exploratory approach may identify additional unexpected patterns at the network level without making prior assumptions about functional localization.

### Rightward asymmetry of motor circuit functional connectivity is associated with motor performance in children with ASC

This study is the first to report a relationship between autistic symptoms and hemispheric lateralization outside the language domain in individuals with ASC; namely, the more rightward asymmetry of the functional correspondence within the motor circuit, the poorer motor performance on three different measures of the PANESS. Both Nielsen et al. [46], who reported atypical lateralization in a language-related network, and Cardinale et al. [45], who reported rightward lateralization of the motor network, did not find such brain-behavioural association. Here, we highlight the importance of their findings by showing functional relevance in children with ASC. Lateralization has been linked to fine motor skills, primarily in relation to handedness. Interestingly, here, we showed that, in addition to fine motor skills, gross motor performances such as gait, balance, timing and sequencing of movements were also associated with atypical hemispheric specialization in children with ASC. Therefore, rightward lateralization may underlie gross motor deficits and atypical gait commonly found in individuals with ASC.

Leftward lateralization of the same circuit has previously been shown to be associated with better motor performance in typically developing children [4]; however, we did not replicate this result here. It remains to be established whether this is due to differing sample characteristics and methods (note that Barber et al. used a different registration approach and laterality index) and whether lateralization of this network constitutes the biological underpinnings for typical motor development or for motor deficits in ASC specifically.

Lateralization in the motor system is present in both structure and function, although it is generally less pronounced compared to the language system lateralization, which would explain why typical children show a subtle shift only towards left here. Structurally, this is characterized by a deeper left central sulcus, increased leftward neuropil in BA4 [76] and expansion of the left hand motor cortex in right-handers [77]. Guye et al. [78] show that whole-brain functional connectivity is more extensive with the left than with the right primary motor cortex. Left premotor and parietal regions are more strongly implicated in higher order actions [79]. Also, the planning of complex, sequential movements [79–81], bimanual coordination [82, 83] and response selection [84] have been attributed to the left hemisphere, which is interesting considering the problems individuals with ASC experience with fine motor skills [85] and planned sequencing of actions [86]. In contrast, sensory processing and spatial aspects of sensorimotor actions have been reported to show right hemisphere dominance [87–89]. It has been suggested that posture and limb position [90], as well as the use of proprioceptive feedback [91, 92], are preferentially lateralized to the right hemisphere in right-handed individuals. Interestingly, we have previously reported that individuals with ASC rely to a much greater extent on proprioceptive feedback during motor learning, which is related to more severe motor impairments and other social symptoms [93]. Atypical rightward lateralization of motor circuits might constitute a neural mechanism contributing to this finding.

Reports of disturbance in establishing typical patterns of hemispheric specialization in ASC date back to the consistently replicated observation of increased rates of left-handedness among individuals with ASC [94–101]. Left-handers activate the right premotor cortex during contra- and ipsilateral finger movements [102] and have increased intrasulcal surface in the right precentral gyrus [76]. Interestingly, our results are not driven by differences in handedness, as participants were matched on a measure of handedness and were right-handed. Still, it will be interesting to explore whether the observed pattern is even more pronounced in left-handed individuals with ASC.

### Implications of motor deficits in ASC

Motor deficits are present in at least 80 % of children with ASC [28, 103, 104] comprising some of the earliest signs, such as delays in motor milestones [105, 106]. Later on in development, motor-related deficits persist and become more apparent, with deficits in gross and fine motor coordination [19, 47, 107, 108], atypical gait and posture [109, 110] and particularly difficulty with performance of skilled gestures (i.e. praxis) [18, 27, 28].

In line with this, it has been argued that core social and communicative symptoms in autism might be subserved by the same neural systems underpinning motor-related dysfunction [111, 112]. In fact, there is a close relationship between motor and social-communicative behaviours: motor development is the prerequisite for speech, articulation and engagement in the social world through directing attention, grasping and sharing things, and approaching, imitating and responding to others. In line with this, Iverson and Braddock [113] have shown that children with language deficits have poorer fine and gross motor skills. At the same time, production and understanding of actions, such as performance of sequences of actions and following verbal commands, are dependent on the development of language skills. Thus, atypical lateralization in related cognitive domains such as language might be influenced by the same developmental mechanisms [114, 115]. Notably, here, we did not find a significant association between atypical motor circuit lateralization and social-communicative sub-measures of either the ADOS or ADI. Given that other studies find such relationship [116], future research needs to examine a potential association with more specific measures of atypical social development in ASC and explore the link between motor and social networks directly at the neuronal level.

### Limitations and future directions

Here, we report atypical laterality of the motor network in children with ASC. Given that ASC is a neurodevelopmental condition, it remains to be established whether the observed pattern is stable across the lifespan. Many studies show temporal test-retest reliability and reproducibility of functional resting-state networks [117–119]. It has been argued that neural coupling configurations are dynamic and transient over time with fluctuations in connection strengths [120, 121]. As arousal, sleep [122], conscious [123], cognitive [124] and emotional [125] states constitute influencing factors, only replication in independent, longitudinal samples can confirm the stability of the reported findings. Age-related changes have been described in a range of different networks, with decreasing connectivity representing increasing hemispheric specialization and increasing connectivity representing increasing functional cooperation [126]. The same authors also reported not only linear but also quadratic and cubic age effects on changes of functional connectivity, emphasizing the need for longitudinal samples to fully explore the stability and plausible developmental trend of the reported findings.

One limitation concerns the heterogeneity within ASC. Children with ASC frequently have other comorbid conditions, such as ADHD, which is another condition associated with atypical lateralization [127]. Also, Mahajan et al. [128] showed that individuals with ASC with and without ADHD show differences in motor cortex laterality with children with ASC without ADHD exhibiting rightward increases in M1, whereas children with ASC and comorbid ADHD showing left-lateralized increases. Medication exposure is another possible confound which might influence connectivity strength [129–131]. Overall, a large portion of individuals with ASC have psychiatric comorbidities during their lifespan (ADHD 28–44 %; anxiety 42–56 %; depression 12–70 %; oppositional defiant disorder 16–28 %; [132]) and around 70 % of children with ASC use some form of psychoactive medication [133, 134], making any sample that excludes these individuals less representative of the autistic population.

It remains to be established whether these results are restricted to lateralized left hemisphere functions or whether right hemisphere functions such as visuospatial abilities are also affected. Cardinale et al. [45] report rightward asymmetries of more widespread networks including those underlying visuospatial attention, but no other studies have looked at hemispheric specialization of visuospatial information processing in ASC. Future research should explore whether stronger rightward lateralization might underpin superior visual attention and weak central coherence in ASC and whether this potentially differentiates between individuals with ASC with and without enhanced visuospatial processing abilities.

Heterogeneity within the autism spectrum is the biggest challenge in the current research. It will be of interest to explore whether atypical motor network lateralization is characteristic of an as-yet undefined subgroup within ASC, such as language delay, for example, [60]. Other factors constituting heterogeneity include handedness, sex or IQ. We previously showed that handedness can be a marker of rightward lateralization of structural connectivity of regions connecting sensorimotor cortical areas [135]. Whether this also applies to functional lateralization remains to be established. As for sex, our findings here were most pronounced when including both males and females. Sex-stratified analyses show an overlapping functional organization in males and females with ASC regarding lateralization of the motor network. In general, few studies have looked at sex differences in the laterality of resting-state connectivity. Tian et al. [136] reported that males show more locally efficient right hemisphere networks, whereas females have more locally efficient left hemisphere networks. Zuo et al. [126] reported that homotopic resting-state connectivity in the dorsolateral prefrontal cortex and the amygdala show opposite developmental trajectories in males and females. How functional connectivity and its lateralization further differ across genders in ASC awaits investigation. Regarding IQ, our sample was only matched on perceptual reasoning IQ; however, additional analyses showed that results remained largely unaffected by full-scale IQ (i.e. when controlling for FIQ). As motor delay is mostly evident in low-functioning individuals [137, 138], it will be important to explore whether this result is more pronounced in this sub-population with ASC.

The present findings were based on patterns observed in the so-called *resting state*, and it will be interesting to explore whether atypical rightward lateralization remains robust under conditions when the motor execution network is active, as well as whether they are more pronounced in particular parcels underlying the motor homunculus. Lastly, here, we focused on atypical intrahemispheric connectivity. However, many studies have shown that atypical interhemispheric connectivity (such as reduced corpus callosum size) contributes to atypical connectivity in ASC [139–141]. Efficient information processing across the whole brain substantially depends on interhemispheric integration, which may be impaired in ASC [142]. Herbert et al. [143] show that only intrahemispheric connectivity is impaired in ASC, whereas others demonstrate that intra- and interhemispheric connectivities are both affected [141, 144]. Particularly, the right hemisphere regions involved in visuospatial processing have been suggested to rely on more integrative, interhemispheric processing [5]. Thus, future studies need to explore whether atypical lateralization in ASC extends to cross-hemispheric networks, particularly for functions such as visuospatial skills.

## Conclusions

Our novel findings show that atypical functional lateralization in ASC extends beyond the language domain to functional circuits underlying motor execution. The deficit in children with ASC in establishing a typical pattern of hemispheric specialization for motor control may contribute to difficulties with motor skill development and might even form the early basis for the development of social and communicative impairments. It remains to be established whether atypical lateralization in functionally related domains (motor and language functions) share common neurodevelopmental origins and trajectories.

## Abbreviations

AC, anterior cerebellum; ADI-R, Autism Diagnostic Interview–Revised; ADOS, Autism Diagnostic Observation Schedule; BOLD, blood-oxygen-level-dependent; ASC, autism spectrum conditions; CSF, cerebrospinal fluid; DMN, default mode network; EHI, Edinburgh Handedness Inventory; FD, framewise displacement; FIQ, full-scale IQ; GSR, global signal regression; LH, left hemisphere; LI, laterality index; PANESS, Physical and Neurological Examination of Subtle Signs; PRI, perceptual reasoning index; PUT, putamen; RH, right hemisphere; SMA-d, bilateral supplementary motor area-dorsal segment; SMA-r, bilateral supplementary motor area-rostral segment; SMC, sensorimotor cortex; TH, thalamus; VCI, verbal comprehension index; WM, white matter

## Additional file

Additional file 1: Group differences in LIs of individual DMN connections and group differences in LIs of individual visual network connections. (DOCX 83 kb)
