# Supplementary material for: Atypical lateralization of motor circuit functional connectivity in children with autism is associated with motor deficits
Source: Mol Autism. 2016 Jul 14;7:35. doi: 10.1186/s13229-016-0096-6 (PMC4946094; doi:10.1186/s13229-016-0096-6)
Supplement: Additional file 1: — Group differences in LIs of individual DMN connections and group differences in LIs of individual visual network connections. (DOCX 83 kb) [file 13229_2016_96_MOESM1_ESM.docx]

| Characteristics | ASC (n=44)  Mean (SD) [Range] | Controls (n=80)  Mean (SD) [Range] | Statistics |
| --- | --- | --- | --- |
| Gender  NoLD (ASP)  LD (HFA)  Age^a^ | 36M; 8F  21M; 6F  15M; 2F  10.23 (1.51) [8.01-12.99] | 58M; 22F  -  -  10.15 (1.08) [8.07-12.76] | ns  -  -  ns |
| Full-Scale IQ^b^ | 104.30 (14.76) [73-141] | 113.10 (10.59) [85-140] | Con>ASC |
| VCI^b^ | 107.39 (15.40) [79-134] | 118.00 (12.49) [85-140] | Con>ASC |
| PRI^a^  Handedness^a^  ADI-R^c^  Social  Communication  RSB  ADOS-G^c^  Communication  Social  RSB | 107.34 (13.99) [79-135]  85.86 (14.60) [50-100]  20.52 (5.99) [10-30]  15.89 (4.79) [4-25]  6.61 (2.15) [3-12]  3.36 (1.10) [1-7]  7.45 (1.98) [4-12]  3.00 (1.67) [0-6] | 109.29 (11.61) [79-133]  84.96 (16.59) [41-100]  -  -  -  -  -  - | ns  ns  -  -  -  -  -  - |

Abbreviations: LD= individuals with ASC with language delay; NoLD= individuals with ASC without language delay; PRI= Perceptual Reasoning Index; VCI: Verbal Comprehension Index;

a) There were no significant differences between the ASC and control groups in age, sex, PRI, or handedness (*p*>.05).

b) The two groups significantly differed in FIQ and VCI (*p*<0.001).

c) Information was available for all 44 individuals with ASC

**Group differences in LIs of individual DMN connections**

| Connection | *F* (1,112) | *p* | *q* |
| --- | --- | --- | --- |
|  |  |  |  |
| PCC-RSC | 1.206 | 0.275 | 0.525 |
| PCC-IFG | 1.691 | 0.196 | 0.457 |
| PCC-LP | 3.427 | 0.067 | 0.302 |
| PCC-MPFC | 2.768 | 0.099 | 0.302 |
| PCC-PHG | 0.094 | 0.76 | 0.792 |
| PCC-SFG | 0.561 | 0.455 | 0.714 |
| RSC-IFG | 0.428 | 0.515 | 0.721 |
| RSC-LP | 2.968 | 0.088 | 0.302 |
| RSC-MPFC | 0.206 | 0.651 | 0.721 |
| RSC-PHG | 3.901 | 0.051 | 0.302 |
| RSC-SFG | 0.512 | 0.476 | 0.714 |
| IFG-LP | 3.23 | 0.075 | 0.302 |
| IFG-MPFC | 7.371 | 0.008 | 0.168 |
| IFG-PHG | 0.204 | 0.652 | 0.721 |
| IFG-SFG | 2.648 | 0.106 | 0.302 |
| LP-MPFC | 0.239 | 0.626 | 0.721 |
| LP-PHG | 1.26 | 0.264 | 0.525 |
| LP-SFG | 0.234 | 0.63 | 0.721 |
| MPFC-PHG | 2.518 | 0.115 | 0.302 |
| MPFC-SFG | 0.07 | 0.792 | 0.792 |
| PHG-SFG | 0.618 | 0.434 | 0.714 |

Abbreviations: posterior cingulate cortex (PCC), medial prefrontal cortex (MPFC), retro-splenial cortex (RSC), lateral parietal cortex (LP), superior frontal gyrus (SFG), inferior frontal gyrus (IFG), parahippocampal gyrus (PHG).

**Group differences in LIs of individual Visual Network connections**

| Connection | *F* (1,112) | *p* | *q* |
| --- | --- | --- | --- |
|  |  |  |  |
| ExC-ExP | 1.222 | 0.271 | 0.567 |
| ExC-V1c | 0.16 | 0.69 | 0.871 |
| ExC-V1p | 0.056 | 0.813 | 0.871 |
| ExC-V3cv | 1.543 | 0.217 | 0.567 |
| ExC-V3pv | 1.02 | 0.315 | 0.567 |
| ExP-V1c | 1.275 | 0.261 | 0.567 |
| ExP-V1p | 0.128 | 0.721 | 0.871 |
| ExP-V3cv | 2.393 | 0.125 | 0.567 |
| ExP-V3cp | 0.346 | 0.558 | 0.837 |
| V1c-V1p | 0.066 | 0.798 | 0.871 |
| V1c-V3cv | 0.015 | 0.903 | 0.871 |
| V1c-V3pv | 2.304 | 0.132 | 0.567 |
| V1p-V3cv | 0.919 | 0.34 | 0.567 |
| V1p-V3pv | 1.84 | 0.178 | 0.567 |
| V3cv-V3pv | 3.371 | 0.069 | 0.567 |

Abbreviations: central and peripheral primary visual cortex (V1_c_ and V1_p_), the central and peripheral regions near visual area V3_v_ (V3_pv_ and V3_cv_) and the extrastriate regions of the central and peripheral visual subnetworks (ExP and ExC).
